# Supplementary material for: Identification of photoperiod-induced specific miRNAs in the adrenal glands of Sunite sheep (Ovis aries)
Source: Front Vet Sci. 2022 Jul 22;9:888207. doi: 10.3389/fvets.2022.888207 (PMC9354845; doi:10.3389/fvets.2022.888207)
Supplement: Supplementary file 1 [file Data_Sheet_1.ZIP › Supplementary file/miRDeep2 file.zip!/miRDeep2 file/Know_Structure/SPLP42A1/oar-mir-191.pdf]

miRBase precursor : oar-mir-191
Total read count : 93326
oar-miR-191 read count : 93086
remaining reads : 240

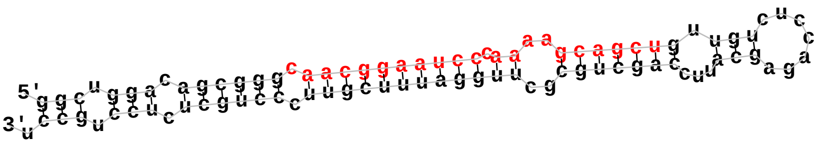

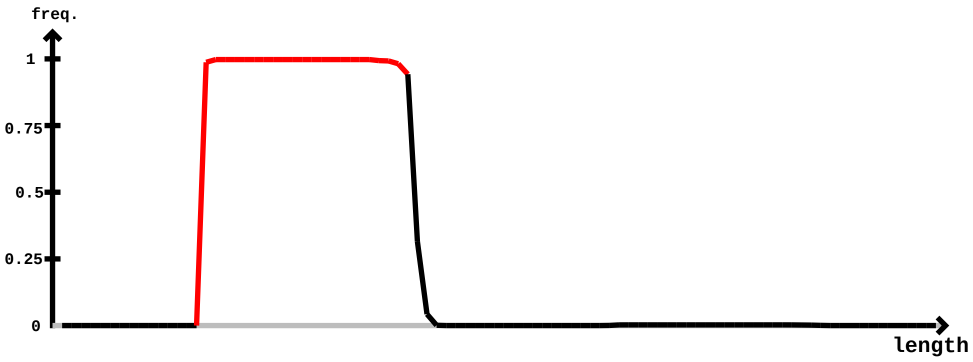


```
              oar-miR-191
5'- ggcuggacagcgggcaacggaaucccaaaagcagcuguugucuccagagcauuccagcugcgcuuggauuucguucccugcucuccugccu  -3'    exp
    (((.(((.((((((.((((((((((.((..(((((((.(((.......)))...)))))))..)))))))))))).)))))).))).))).   reads      mm      sample
    ..............caacggaaucccaaaagc...........................................................   365        0       seq
    ..............caacggaaucccaaaagca..........................................................   118        0       seq
    ..............caacggaaucccaaaagcag.........................................................   977        0       seq
    ..............caacggaaucccaaaagcagc........................................................   3640       0       seq
    ..............caacggaaucccaaaagcagcu.......................................................   58234      0       seq
    ..............caacggaaucccaaaagcagcug......................................................   25024      0       seq
    ..............caacggaaucccaaaagcagcugu.....................................................   3675       0       seq
    ..............caacggaaucccaaaagcagcuguu....................................................   96         0       seq
    ..............caacggaaucccaaaagcagcuguug...................................................   1          0       seq
    ...............aacggaaucccaaaagca..........................................................   1          0       seq
    ...............aacggaaucccaaaagcag.........................................................   4          0       seq
    ...............aacggaaucccaaaagcagc........................................................   28         0       seq
    ...............aacggaaucccaaaagcagcu.......................................................   301        0       seq
    ...............aacggaaucccaaaagcagcug......................................................   400        0       seq
    ...............aacggaaucccaaaagcagcugu.....................................................   214        0       seq
    ...............aacggaaucccaaaagcagcuguu....................................................   4          0       seq
    ................acggaaucccaaaagcagcu.......................................................   2          0       seq
    ................acggaaucccaaaagcagcug......................................................   1          0       seq
    ................acggaaucccaaaagcagcugu.....................................................   1          0       seq
    .......................................................agcugcgcuuggauuucguuc...............   1          0       seq
    .......................................................agcugcgcuuggauuucguucc..............   1          0       seq
    ........................................................gcugcgcuuggauuucguu................   1          0       seq
    ........................................................gcugcgcuuggauuucguuc...............   13         0       seq
    ........................................................gcugcgcuuggauuucguucc..............   14         0       seq
    ........................................................gcugcgcuuggauuucguuccc.............   11         0       seq
    ........................................................gcugcgcuuggauuucguucccu............   3          0       seq
    .........................................................cugcgcuuggauuucguu................   7          0       seq
    .........................................................cugcgcuuggauuucguuc...............   24         0       seq
    .........................................................cugcgcuuggauuucguucc..............   19         0       seq
    .........................................................cugcgcuuggauuucguuccc.............   83         0       seq
    .........................................................cugcgcuuggauuucguucccu............   57         0       seq
    .........................................................cugcgcuuggauuucguucccug...........   2          0       seq
    ..........................................................ugcgcuuggauuucguucccu............   4          0       seq
```
